# Sequential PD-1 inhibitor after adjuvant radiotherapy for postoperative oral cancer: a propensity score matching retrospective cohort study

**DOI:** 10.3389/fonc.2026.1798895

**Published:** 2026-05-14

**Authors:** Shaoqing Chen, Ping Xie, Runhong Mei, Mingbin Hu, Yi Li, Xiongming Zhou, Jianwei Liu, Chongchong Zhang, Shanshan Wang, Yeyuan Chen, Xin Xiong, Jie Zhang

**Affiliations:** 1 Department of Oncology, The First Affiliated Hospital, Jiangxi Medical College, Nanchang University, Nanchang, Jiangxi, China; 2 Jiangxi Provincial Key Laboratory of Oral Diseases, The First Affiliated Hospital, Jiangxi Medical College, Nanchang University, Nanchang, Jiangxi, China; 3 Department of Stomatology, The First Affiliated Hospital, Jiangxi Medical College, Nanchang University, Nanchang, Jiangxi, China; 4 Department of Pathology, The First Affiliated Hospital, Jiangxi Medical College, Nanchang University, Nanchang, China; 5 Department of Radiology, The First Affiliated Hospital, Jiangxi Medical College, Nanchang University, Nanchang, China

**Keywords:** adjuvant therapy, combination therapy, oral cancer, PD-1 inhibitor, postoperative high-risk factors

## Abstract

**Objective:**

To explore the efficacy and safety of adjuvant radiotherapy and sequential programmed cell death protein-1 (PD-1) inhibitors in high-risk patients after oral cancer surgery.

**Methods:**

This single-center, retrospective study included high-risk oral cancer patients who received adjuvant therapy at The First Affiliated Hospital of Nanchang University from January 2013 to December 2024. Patients were divided into a PD-1 inhibitor group and a control group based on whether they received PD-1 inhibitor therapy. Propensity Score Matching (PSM) was used to match patients 1:1. Overall Survival (OS) and Adverse Events (AEs) were compared between the PD-1 inhibitor group and the control group.

**Results:**

A total of 262 patients were included in this study, comprising 125 in the PD-1 inhibitor group and 137 in the control group. After PSM, 89 patients from the PD-1 inhibitor group were successfully matched with 89 patients from the control group. The risk of death in the PD-1 inhibitor group was significantly lower than in the control group, with a Hazard Ratio (HR) of 0.54 (95% confidence interval [CI] 0.30-0.98), P = 0.04. The median OS was not reached (NR) in the PD-1 inhibitor group, whereas it was 55.0 months (95% CI, 48.2–63.5) in the control group. The incidence of adverse events in the PD-1 inhibitor group was higher than in the control group. The most common adverse reactions in the PD-1 inhibitor group were gastrointestinal reactions (40.0%), myelosuppression (31.2%), and oral mucositis (27.2%), all of which were higher than in the control group (4.4%, 5.1%, and 0%, respectively). Adverse events exclusive to the combination group included oral mucositis, radiation dermatitis (8.8%), and hepatitis. One death (0.7%) was reported in the monotherapy group, while there were no deaths in the combination group.

**Conclusion:**

In the adjuvant treatment of high-risk postoperative oral cancer patients, sequential PD-1 inhibitor can significantly improve patient survival benefits, but the incidence of adverse events is higher.

## Introduction

Head and neck cancer is the seventh most common malignancy worldwide, with over 90% being squamous cell carcinoma. Oral Squamous Cell Carcinoma (OSCC), which occurs in the oral mucosa, is a particularly common subtype of Head and Neck Squamous Cell Carcinoma (HNSCC) (1). OSCC is associated with a poorer prognosis compared to other head and neck squamous cell carcinomas. Global cancer statistics for 2020 show that there are over 377,713 new cases annually (2). However, about 60% of patients are already in a locally advanced stage at their initial diagnosis (3, 4). For patients with high-risk factors for postoperative recurrence, the standard treatment is radical surgery combined with adjuvant chemoradiotherapy (5, 6), but the five-year overall survival rate is only 50% (4, 7), necessitating better treatment strategies.

In recent years, immune checkpoint inhibitors targeting programmed cell death protein-1 (PD-1) have made breakthrough progress in the treatment of solid tumors (8–10). Clinical research data indicate that PD-1 inhibitors, represented by nivolumab, can significantly reduce the risk of death in patients with recurrent/metastatic HNSCC by 32%. More notably, this treatment has increased the 2-year overall survival rate from 6.0% with traditional chemotherapy to 16.9% (11, 12). The pivotal Phase III clinical trial KEYNOTE-048 showed that in the population with PD-L1 CPS≥1, the median duration of response in the pembrolizumab monotherapy group reached 23.4 months (95% confidence interval [CI] 7.5-NR), which was significantly longer than the 4.5 months (95% CI 4.1–5.6) in the standard cetuximab group (hazard ratio [HR]=0.49, 95% CI 0.33–0.73) (13). This evidence-based medicine has established the important role of PD-1 inhibitors in the systemic treatment of recurrent/metastatic HNSCC.

In the field of adjuvant therapy, the synergistic effect of combining immune checkpoint inhibitors with chemotherapy has shown significant clinical benefits in multiple types of cancer (14, 15). Studies have shown that PD-1/PD-L1 inhibitors can significantly extend disease-free survival (DFS) by activating the T-cell immune response in the postoperative tumor microenvironment (16). For example, in the IMpower010 trial, adjuvant atezolizumab increased the 3-year DFS rate for patients with stage II-IIIA non-small cell lung cancer from 48.2% in the traditional treatment group to 60.0% in the atezolizumab group. However, there is limited evidence for adjuvant therapy in high-risk postoperative head and neck tumors or oral cancer (17, 18).

This retrospective study uses the Propensity Score Matching (PSM) method to balance baseline characteristics between groups to explore the efficacy and safety of sequential PD-1 inhibitor with adjuvant therapy in high-risk postoperative oral cancer patients.

## Methods

### Study design and population

This was a single-center retrospective cohort study that included oral cancer patients who received sequential PD-1 inhibitor therapy after adjuvant therapy at The First Affiliated Hospital of Nanchang University from January 2013 to December 2024. The study was approved by the Clinical Research Ethics Committee of The First Affiliated Hospital of Nanchang University (Ethics Approval No.: ISL20250313), and the requirement for patient informed consent was waived. The study adhered to the Declaration of Helsinki, and no patients were enrolled in clinical trials sponsored by pharmaceutical companies.

Inclusion criteria were:

1. histologically or cytologically confirmed oral cancer;
2. age ≥18 years;
3. Eastern Cooperative Oncology Group (ECOG) performance status of 0–1;
4. completion of curative-intent surgery with postoperative pathological high-risk features, defined as the presence of one or more of the following: AJCC 8th edition stage III–IV disease, lymph node metastasis, or positive/close surgical margins (≤5 mm);
5. availability of complete clinical and follow-up data.

Patients were classified into the PD-1 inhibitor group or the control group according to whether they received PD-1 inhibitor after adjuvant therapy.

### Procedures

All patients received intensity-modulated radiotherapy (IMRT) delivered by experienced head and neck oncology teams. Target delineation and treatment planning followed National Comprehensive Cancer Network (NCCN) guidelines. Postoperative high-risk regions, including the tumor bed and high-risk nodal drainage areas, received 60 Gy, while low-risk nodal areas received 54 Gy, using conventional fractionation. The use of concurrent cisplatin-based chemoradiotherapy was determined by pathological risk factors. Patients with positive surgical margins or extranodal extension received concurrent chemoradiotherapy, with cisplatin administered at 75 mg/m² intravenously on day 1 and day 22 of radiotherapy. Other postoperative high-risk patients received radiotherapy alone. In the experimental group, patients received adjuvant PD-1 inhibitor–based systemic therapy after radiotherapy or chemoradiotherapy. PD-1 inhibitors were administered intravenously every 3 weeks, with zimberelimab being the most frequently used agent. PD-1 inhibitors were given sequentially or concurrently with chemotherapy for a total of four cycles following completion of radiotherapy or chemoradiotherapy. In the control group, patients received standard adjuvant therapy consisting of chemotherapy alone after radiotherapy or chemoradiotherapy, without PD-1 inhibitors. The chemotherapy regimen and number of cycles were identical to those in the experimental group, with a total of four cycles administered. Routine monitoring during treatment included complete blood counts and liver, renal, and thyroid function tests. Radiologic assessments were performed after every two cycles of PD-1 inhibitor therapy. PD-1 inhibitors were withheld and corticosteroids initiated for grade ≥3 immune-related adverse events. For grade ≥3 radiotherapy- or chemoradiotherapy-related toxicities, chemotherapy dose modification or temporary interruption of radiotherapy was permitted. Treatment was discontinued upon disease progression or unacceptable toxicity, after which control-group patients received subsequent systemic therapy or best supportive care. All treatment decisions were made by a multidisciplinary team in accordance with NCCN guidelines and individual patient characteristics, and all procedures followed standardized clinical practice.

### Follow-up and assessment

This study established clear data definitions [e.g., start and end times for OS, criteria for determining adverse events (AEs)], unified inclusion criteria for the high-risk population (pathological details such as positive margins, extranodal extension), and developed standardized efficacy evaluation protocols based on RECIST 1.1 criteria. Patients underwent standardized assessments before each PD-1 inhibitor infusion and during radiotherapy. Two independent radiologists with over 5 years of experience in head and neck tumor imaging diagnosis assessed tumor response from follow-up imaging. Disagreements were resolved by a senior radiologist.

Treatment safety was continuously assessed through regular laboratory tests, vital sign monitoring, and clinical symptom documentation. Adverse events were analyzed as all adverse events occurring during the treatment period. The follow-up deadline for the study was February 2025, and follow-up included clinical examinations, imaging assessments, and adverse reaction evaluations.

### Endpoints

The primary endpoint of this study was Overall Survival (OS), defined as the time from the date of surgery to death from any cause. The secondary endpoint was AE. Covariates for survival analysis included: age, sex, Eastern Cooperative Oncology Group (ECOG), Tumor Stage (T stage), Node Stage (N stage), Chemotherapy (Chemo), Pathological Differentiation, Margin Status (Margin), Perineural Invasion (PNI), Lymphovascular invasion (LVI).

### Statistical analysis

This study employed a 1:1 PSM analysis. The nearest neighbor method was used for matching, with a caliper value set at 0.2. Furthermore, the balance of variables before and after PSM was validated using standardized mean difference (SMD) analysis. Categorical variables were analyzed using the chi-square test or Fisher’s exact test, and continuous variables were analyzed using the Mann-Whitney U test or t-test, depending on their distribution. The Kaplan-Meier method and the Log-rank test were used to compare OS between the two groups. Univariate and multivariate Cox proportional hazards models were used to assess the independent impact of the combination therapy on OS, based on both the matched sample and the unmatched full population. Additionally, a sensitivity analysis was performed by incorporating smoking history, alcohol consumption, and betel nut habit into the multivariate Cox model to confirm the robustness of the treatment effect. Subgroup analyses were performed based on pre-specified clinically relevant parameters. A two-sided p<0.05 was considered significant. Statistical analysis was performed using R 4.4.3.

## Results

### Patient baseline characteristics

**Figure 1:**
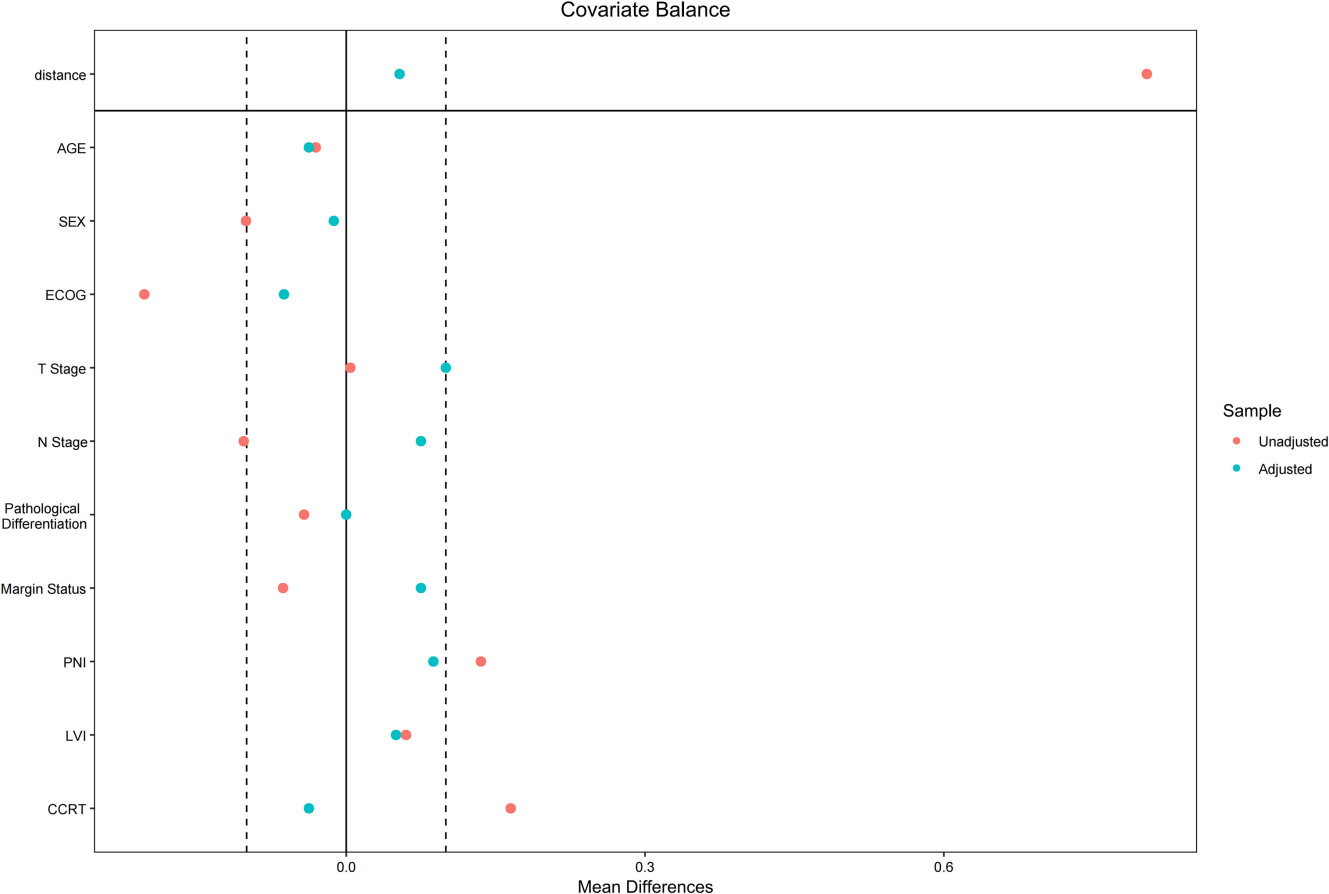
Standardized mean differences of baseline covariates before and after propensity score matching. ECOG, Eastern Cooperative Oncology Group; T Stage, tumor stage; N Stage, node stage; PNI, perineural invasion; LVI, lymphovascular invasion; CCRT, concurrent chemoradiotherapy; OS, overall survival; PSM, propensity score matching.

**Figure 2:**
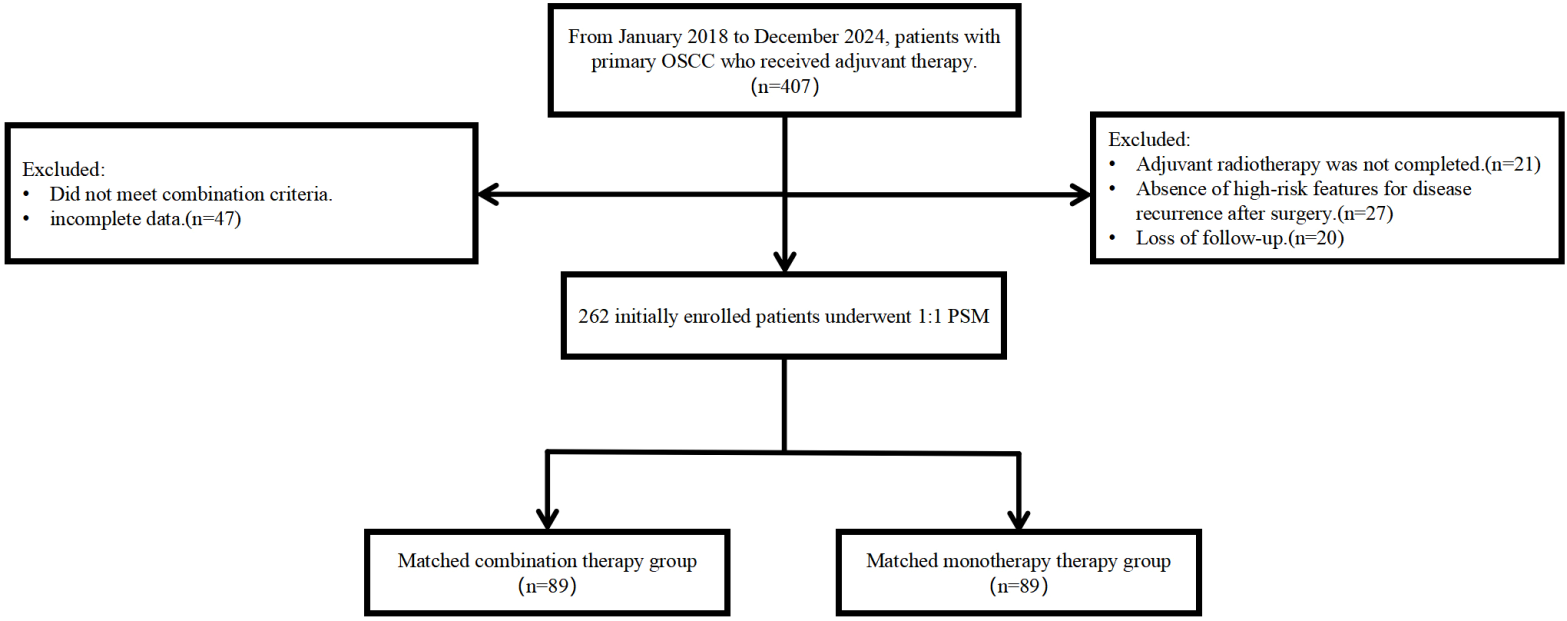
Patient selection flowchart.

During the study period, 262 oral cancer patients from The First Affiliated Hospital of Nanchang University were included, with 125 in the PD-1 inhibitor group and 137 in the control group. Before matching, 60.0% (75/125) of patients in the PD-1 inhibitor group were <60 years old, compared to 56.9% (78/137) in the control group. Males constituted 81.6% (102/125) of the PD-1 inhibitor group and 71.5% (98/137) of the control group. After PSM, 89 patients from the PD-1 inhibitor group were successfully matched with 89 patients from the control group. There were no significant differences in baseline characteristics between the two groups after matching (**Figure 1**). In the PD-1 inhibitor group, 48 patients were <60 years old, and 80.9% (72/89) were male. In the control group, 49 patients were <60 years old, and 78.7% (70/89) were male. Detailed patient baseline characteristics are shown in **Table 1**, and the patient selection flowchart is in **Figure 2**. Safety was assessed in all 262 patients before matching, with adverse events summarized in **Table 2**.

**Table 1:** Patient baseline characteristics of PD-1 inhibitor group and control group before and after PSM.

Characteristics	Before PSM			After PSM		
	PD-1 inhibitor group (n=125)	Control group (n=137)	p value	PD-1 inhibitor group (n=89)	Control group (n=89)	p value
Age			0.598			0.882
<60	75 (60.0)	78 (56.9)		48 (53.9)	49 (55.1)	
≥60	50 (40.0)	59 (43.1)		41 (46.1)	40 (44.9)	
Sex			0.045			0.723
Female	23 (18.4)	39 (28.5)		17 (19.1)	19 (21.3)	
Male	102 (81.6)	98 (71.5)		72 (80.9)	70 (78.7)	
ECOG			0.001			1.000
0	81 (64.8)	61 (44.5)		50 (56.2)	50 (56.2)	
1	44 (35.2)	76 (55.5)		39 (43.8)	39 (43.8)	
Tumor Stage			0.948			0.651
1&2	67 (53.6)	74 (54.0)		53 (59.6)	50 (56.2)	
3&4	58 (46.4)	63 (46.0)		36 (40.4)	39 (43.8)	
Node Stage			0.111			0.287
0&1	74 (59.2)	67 (49.8)		45 (50.6)	52 (58.4)	
2&3	51 (40.8)	70 (51.1)		44 (49.4)	37 (41.6)	
Pathological Differentiation			0.459			0.754
G1	50 (40.0)	49 (35.8)		37 (41.6)	39 (43.8)	
G2-G3	75 (60.0)	88 (64.2)		52 (58.4)	50 (56.2)	
Margin Status			0.201			1.000
Negative	101 (80.8)	102 (74.5)		69 (77.5)	69 (77.5)	
Positive	24 (19.2)	35 (25.5)		20 (22.5)	20 (22.5)	
Perineural Invasion			0.021			0.294
Present	68 (54.4)	56 (40.9)		51 (57.3)	44 (49.4)	
Absent	57 (45.6)	81 (59.1)		38 (42.7)	45 (50.6)	
Lymphovascular Invasion			0.276			0.428
Present	44 (35.2)	40 (29.2)		34 (38.2)	29 (32.6)	
Absent	81 (64.8)	97 (70.8)		55 (61.8)	60 (67.4)	
Chemo			0.005			0.443
Present	69 (55.2)	53 (38.7)		49 (55.1)	44 (49.4)	
Absent	56 (44.8)	84 (61.3)		40 (44.9)	45 (50.6)	
Smoking history			0.001			0.175
Never	44 (35.2)	78 (56.9)		35 (39.3)	45 (50.6)	
Ever	81 (64.8)	59 (43.1)		54 (60.7)	44 (49.4)	
Alcohol intake			0.541			0.415
Never	86 (68.8)	100 (73.0)		65 (73.0)	59 (66.3)	
Ever	39 (31.2)	37 (27.0)		24 (27.0)	30 (33.7)	
Betel nut habit			0.058			0.396
Never	102 (81.6)	123 (89.8%)		74 (83.1%)	78 (87.6%)	
Ever	23 (18.4)	14 (10.2%)		15 (16.9%)	11 (12.4%)	

PSM, propensity score matching; ECOG, Eastern Cooperative Oncology Group; Chemo, Chemotherapy.

**Table 2:** Adverse events from any cause before matching.

Adverse events	PD-1 inhibitor group (n=125)	Control group (n=137)
GI adverse events	50 (40.0)	6 (4.4)
Myelosuppression	39 (31.2)	7 (5.1)
Oral Mucositis	34 (27.2)	0 (0.0)
Anemia	30 (24.0)	3 (2.2)
Metabolic Abnormalities	15 (12.0)	2 (1.5)
Radiation Dermatitis	11 (8.8)	0 (0.0)
Rash	7 (5.6)	5 (3.6)
Others	7 (5.6)	1 (0.7)
Leukopenia	4 (3.2)	2 (1.5)
Hepatitis	3(2.4)	0 (0.0)
Thrombocytopenia	2 (1.6)	0 (0.0)
Infection	2 (1.6)	0 (0.0)
Atrial Fibrillation	1 (0.8)	0 (0.0)
Death	0 (0.0)	1 (0.7)

Data are presented as n (%). *Numbers represent the highest grades assigned. PD-1, programmed cell death protein 1; GI, gastrointestinal.

### Efficacy

**Figure 3:**
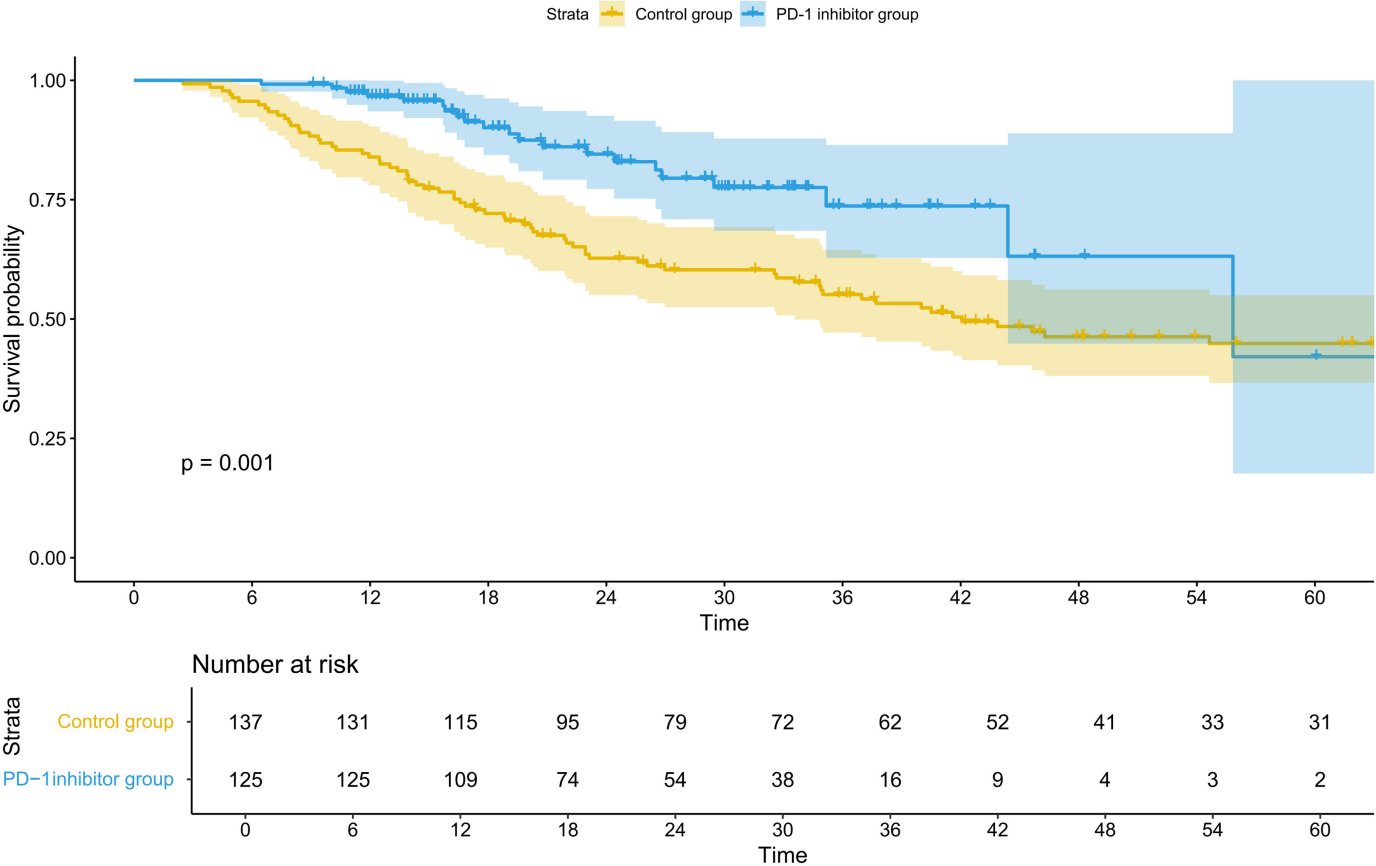
Kaplan–Meier curves of OS before PSM. OS, overall survival; PD-1, programmed cell death protein 1, PSM, propensity score matching.

**Figure 4:**
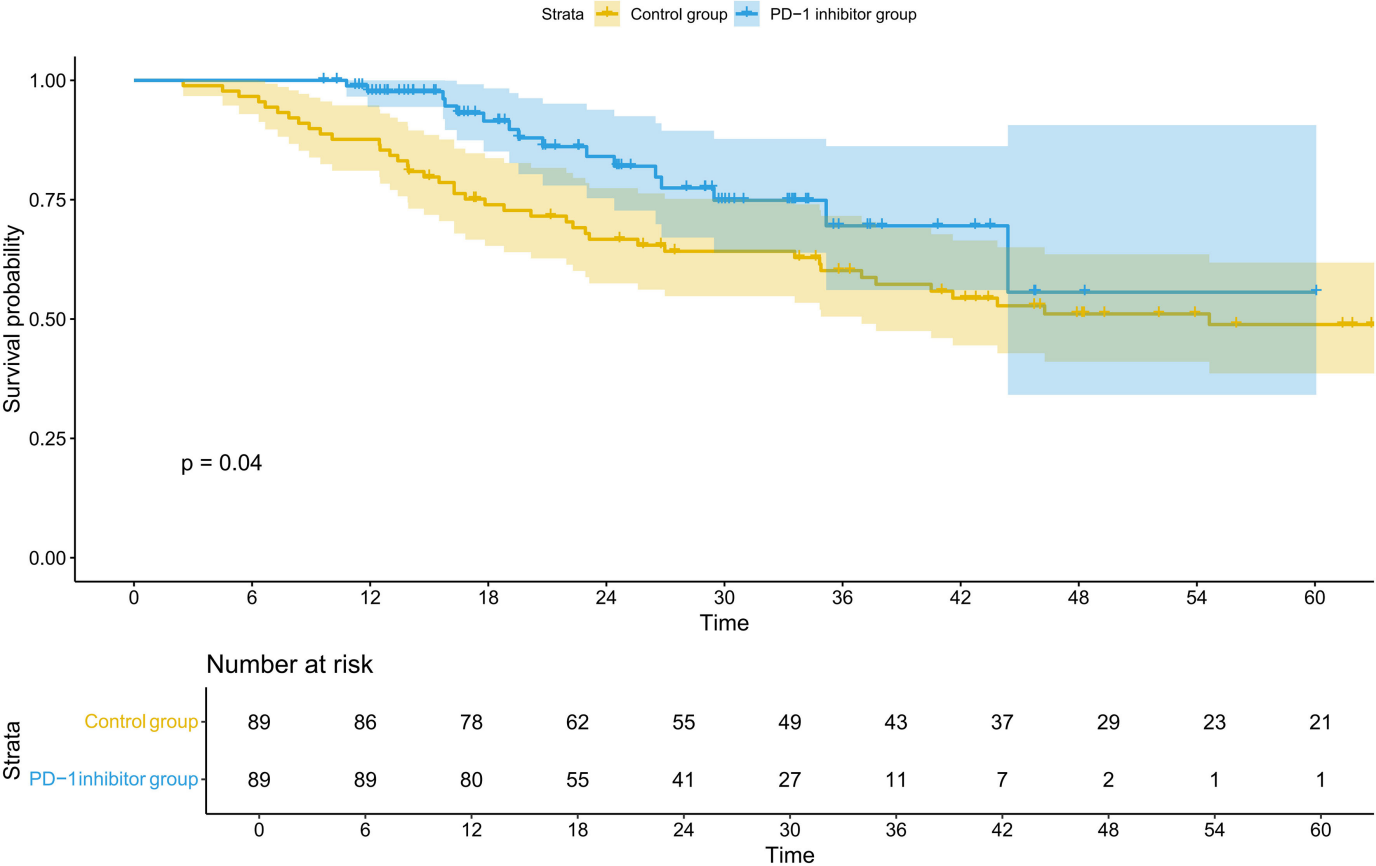
Kaplan–Meier curves of OS after PSM. OS, overall survival; PD-1, programmed cell death protein 1, PSM, propensity score matching.

Before PSM, the OS benefit in the treatment group was significantly better than in the control group (HR = 0.44; 95% CI 0.27–0.73, p=0.001) (**Figure 3**; **Table 3**). After PSM, the OS benefit in the combination therapy group was significantly better than in the control group (HR = 0.54; 95% CI 0.30–0.98, p=0.04) (**Figure 4**; **Table 4**). Compared to the control group, the 5-year OS rate (57.5% vs 50.6%, P = 0.205) and 3-year OS rate (70.8% vs 61.8%, P = 0.366) in the PD-1 inhibitor group showed a numerical improvement, but the difference did not reach statistical significance. However, the median OS was not reached (NR) in the PD-1 inhibitor group, whereas the median OS in the control group was 55.0 months (95% CI, 48.2–63.5).

**Table 3:** Predictors of overall survival before matching.

Parameter	Univariable analysis			Multivariable analysis		
	HR	95% CI	P value	HR	95% CI	P value
Age (<60 vs ≥60)	1.17	0.78-1.77	0.438	0.99	0.61-1.62	0.982
Sex (Female vs. Male)	1.44	0.92-2.24	0.107	1.48	0.93-2.36	0.102
ECOG (1 vs. 0)	1.56	1.04-2.36	0.033	1.38	0.83-2.29	0.213
T Stage (1&2 vs. 3&4)	1.21	0.80-1.82	0.362	1.17	0.77-1.77	0.460
N Stage (0&1 vs. 2&3)	1.64	1.09-2.47	0.018	1.74	1.12-2.69	0.013
Chemo (Present vs. Absent)	0.60	0.39-0.92	0.019	0.68	0.43-1.07	0.092
Pathological differentiation (G1 vs. G2–G3)	0.92	0.61-1.40	0.699	0.80	0.52-1.23	0.313
Margin (Negative vs. Positive)	0.92	0.57-1.49	0.739	0.84	0.52-1.38	0.500
PNI (Present vs. Absent)	0.81	0.53-1.22	0.315	0.64	0.35-1.17	0.147
LVI (Present vs. Absent)	0.97	0.63-1.51	0.904	1.32	0.69-2.54	0.402

ECOG, Eastern Cooperative Oncology Group; T stage, tumor stage; N stage, node stage; Chemo, Chemotherapy; G, grade; margin, margin status; PNI, perineural invasion; LVI, lymphovascular invasion; HR, hazard ratio; CI, confidence interval.

**Table 4:** Predictors of overall survival after matching.

Parameter	Univariable analysis			Multivariable analysis		
	HR	95% CI	P value	HR	95% CI	P value
Age (<60 vs. ≥60)	1.58	0.95-2.64	0.081	1.33	0.69-2.54	0.397
Sex (Female vs. Male)	0.79	0.41-1.52	0.477	0.74	0.37-1.50	0.408
ECOG (1 vs. 0)	1.42	0.85-2.37	0.176	1.37	0.73-2.57	0.325
T Stage (1&2 vs. 3&4)	1.48	0.89-2.48	0.131	1.48	0.87-2.54	0.149
N Stage (0&1 vs. 2&3)	1.33	0.79-2.22	0.280	1.83	1.02-3.29	0.043
Chemo (Present vs. Absent)	0.65	0.39-1.08	0.097	0.74	0.42-1.29	0.286
Pathological differentiation (G1 vs. G2-G3)	0.72	0.43-1.20	0.211	0.72	0.42-1.24	0.237
Margin (Negative vs. Positive)	0.87	0.47-1.61	0.660	0.85	0.45-1.63	0.630
PNI (Present vs. Absent)	0.83	0.50-1.40	0.490	0.717	0.33-1.54	0.394
LVI (Present vs. Absent)	0.94	0.55-1.62	0.831	1.23	0.55-2.73	0.612

ECOG, Eastern Cooperative Oncology Group; T stage, tumor stage; N stage, node stage; Chemo, Chemotherapy; G, grade; margin, margin status; PNI, perineural invasion; LVI, lymphovascular invasion; HR, hazard ratio; CI, confidence interval.

**Figure 5:**
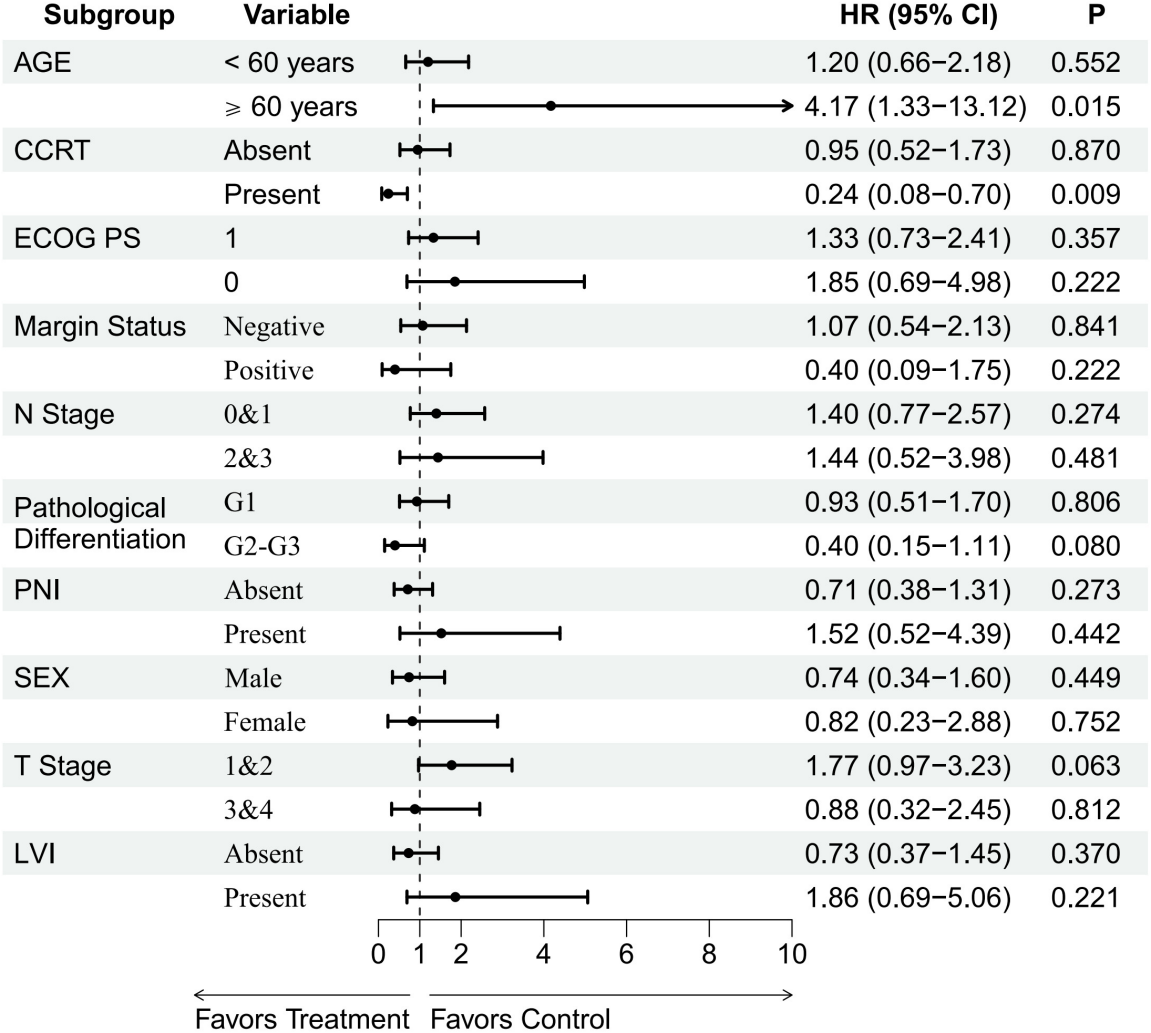
Subgroup analysis of overall survival after matching. CCRT, concurrent chemoradiotherapy; ECOG PS, Eastern Cooperative Oncology Group performance status; N Stage, node stage; G, grade; PNI, perineural invasion; T Stage, tumor stage; LVI, lymphovascular invasion; HR, hazard ratio; CI, confidence interval.

Subgroup analysis (**Figure 5**) showed that patients receiving concurrent chemotherapy with radiotherapy had a significantly lower risk of death (HR = 0.24, 95% CI: 0.08–0.70, p=0.009). Elderly patients (≥60) had a significantly increased risk of death (HR = 4.17, 95% CI: 1.33–13.12, p=0.015). Other variables (Sex, ECOG, T stage, N stage, Pathological Differentiation, PNI, LVI) had p-values >0.05 and did not show statistically significant differences. Furthermore, sensitivity analyses confirmed that the survival benefit of sequential PD-1 inhibitor therapy remained robust and significant (P = 0.038) even after adjusting for potential lifestyle confounders, including smoking, alcohol, and betel nut habits (**Supplementary Tables S2**, **S3**).

### Safety

Safety was assessed in 262 patients (125 in the PD-1 inhibitor group, 137 in the control group). The results showed that the incidence of adverse events in the PD-1 inhibitor plus chemoradiotherapy group was significantly higher than in the chemoradiotherapy alone group. The most common adverse reactions in the PD-1 inhibitor group were gastrointestinal reactions (40.0%), myelosuppression (31.2%), and oral mucositis (27.2%), with incidences significantly higher than in the control group (4.4%, 5.1%, and 0%, respectively). Other major adverse events included anemia (24.0% vs 2.2%), metabolic abnormalities (12.0% vs 1.5%), and radiation dermatitis (8.8% vs 0%). There was one treatment-related death (0.7%) in the control group and no deaths in the PD-1 inhibitor group. The results indicate that while the combination therapy regimen may enhance efficacy, it is associated with a higher risk of severe toxicity, requiring close monitoring, especially of the digestive system, hematopoietic function, and mucosal barriers. Details of adverse events are shown in **Table 2**.

## Discussion

This single-center, retrospective cohort study of patients with oral cancer demonstrates that the addition of a sequential PD-1 inhibitor to standard chemoradiotherapy is associated with a significantly reduced risk of death (HR = 0.54) and a markedly improved median OS (not reached in the treatment group vs. 55.0 months in the control group). These results were confirmed in the PSM-adjusted cohort, suggesting a robust treatment benefit. The prognosis of OSCC is worse than that of other head and neck cancers, a characteristic closely associated with its unique biological features. The dense lymphatic network in the oral cavity leads to a high risk of early lymph node metastasis, while the presence of high-risk factors such as positive surgical margins and extranodal extension significantly contributes to its high recurrence rate and poor prognosis (19). Additionally, the highly immunosuppressive tumor microenvironment prevents the immune system from effectively eliminating cancer cells, making conventional treatment modalities like surgery combined with chemoradiotherapy often insufficient to overcome these challenges (10). This study provides a robust solution to this problem by introducing a sequential PD-1 inhibitor as adjuvant therapy for high-risk OSCC patients. This approach effectively reverses tumor immunosuppression, significantly reducing the risk of death (HR = 0.54) and markedly improving median overall survival. This not only establishes a new paradigm for OSCC treatment but also highlights the critical value and urgent need for precise, individualized adjuvant therapy in improving patients’ long-term survival. However, this survival advantage did not translate into a statistically significant improvement in 3- and 5-year OS rates, despite observed numerical improvements. The combination therapy was associated with a higher rate of adverse events, which were generally manageable. Multivariate analysis identified concurrent chemotherapy during radiotherapy as an independent predictor of longer OS, while advanced age (≥60 years) was an independent predictor of poorer survival.

The biological rationale for combining immunotherapy with chemoradiotherapy (CRT) is compelling. CRT has long been the cornerstone of treatment for locally advanced head and neck cancers, including oral cancer (12, 20). Beyond its direct cytotoxic effects, radiation and certain chemotherapeutic agents are known to induce immunogenic cell death (21, 22). This process leads to the release of tumor-associated antigens and damage-associated molecular patterns (DAMPs), which primes an anti-tumor immune response. Furthermore, radiotherapy can modulate the tumor microenvironment by increasing the expression of MHC class I molecules on tumor cells, promoting T-cell infiltration, and depleting immunosuppressive regulatory T-cells (20, 23). However, this pro-inflammatory shift is often counteracted by the adaptive upregulation of immune checkpoints, particularly the PD-1/PD-L1 axis, which leads to T-cell exhaustion and immune escape (24, 25). By administering a PD-1 inhibitor concurrently with CRT, it is hypothesized that this major resistance mechanism can be overcome, thereby unleashing the full potential of the CRT-induced immune response to achieve more durable tumor control. Our finding that patients receiving concurrent chemotherapy with radiotherapy experienced the most significant survival benefit (HR = 0.24, p=0.009) strongly supports this synergy hypothesis.

The efficacy of PD-1 blockade in recurrent or metastatic HNSCC is well-established (26–28). The landmark KEYNOTE-048 trial established pembrolizumab, alone or with chemotherapy, as a first-line standard of care, demonstrating superior OS over the cetuximab plus chemotherapy. In that trial, pembrolizumab with chemotherapy achieved a median OS of 13.0 months in the total population, with a 5-year OS rate of 16.0% (13). In the setting of recurrence after platinum-based therapy, the CheckMate 141 trial showed that nivolumab monotherapy yielded a median OS of 7.7 months and a 2-year survival rate of 16.9%, superior to standard single-agent chemotherapy (12, 29). Our study, which evaluates the addition of PD-1 inhibitors in the definitive curative-intent setting rather than for recurrent/metastatic disease, shows more promising survival outcomes. The median OS in our PD-1 inhibitor group was not reached and was significantly longer than the control group’s 55.0 months, with a 3-year OS rate of 70.8%. While an indirect comparison, this suggests that introducing immunotherapy earlier in the treatment course, combined with definitive CRT, may yield a greater and more durable survival benefit than its use in later-stage disease. It must be noted, however, that other large randomized trials exploring this combination in locally advanced HNSCC have had mixed results. For example, the JAVELIN Head and Neck 100 trial, which evaluated avelumab (a PD-L1 inhibitor) with CRT, was terminated for futility as it was unlikely to meet its primary endpoint of improving progression-free survival (PFS) (30). This highlights the complexity of timing, patient selection, and the specific checkpoint inhibitor used. The superior outcomes in our study may be attributable to patient-specific factors or the unique biology of oral cavity cancers. In patients with high-risk pathological features, the selection of a 4-cycle consolidation chemotherapy regimen following adjuvant radiotherapy was primarily intended to maximize systemic disease control and eradicate micrometastatic disease. This strategy is consistent with regional clinical practice and existing clinical research evidence (31). A unique feature of our study is the 4-cycle intensive regimen, distinct from the approach in the KEYNOTE-689 trial (32). While KEYNOTE-689 utilized a perioperative strategy involving 2 cycles of neoadjuvant and 15 cycles of adjuvant pembrolizumab to significantly improve event-free survival (32), our 4-cycle strategy prioritized the immediate postoperative window to maximize chemo-immunotherapy synergy. This shorter course was a strategic decision to balance therapeutic intensity with patient compliance and financial feasibility in real-world practice. Despite the reduced duration, our cohort achieved a substantial OS benefit (HR = 0.54), suggesting that this intensive 4-cycle alternative is highly active and practical for high-risk patients in resource-limited settings, warranting further comparison with extended maintenance protocols.

In terms of safety, the addition of a PD-1 inhibitor to CRT unsurprisingly led to a higher incidence of AEs, with 72.0% of patients in the combination arm experiencing an AE versus 24.1% in the control arm. The rate of grade 3–4 AEs was also higher (15.2% vs. 5.1%). The most common AEs in the combination group—gastrointestinal reactions (40.0%), myelosuppression (31.2%), and oral mucositis (27.2%)—are known toxicities of both CRT and immunotherapy. The increased incidence reflects an overlapping and potentially synergistic toxicity profile. While these rates are significant and require diligent clinical management, they appear manageable. The rate of permanent treatment discontinuation due to AEs was low in the PD-1 inhibitor group (2.4%), and there were no treatment-related deaths in this arm. In contrast, one treatment-related death occurred in the standard CRT control group. Notably, the manageable safety profile observed in this study further supports the clinical feasibility of a limited-cycle immunotherapy strategy as an alternative to prolonged maintenance approaches.

The discrepancy between a significant median OS benefit and non-statistically significant 3- and 5-year OS rates is a key finding that warrants discussion. This statistical pattern is common in immunotherapy trials, where a subset of patients derives a profound long-term benefit, creating a “long tail” on the Kaplan-Meier survival curve. This effect can drive a substantial improvement in median survival time even if the overall survival rate at specific time points does not reach statistical significance across the entire cohort. It suggests that while not all patients benefit, those who do may achieve very durable disease control. This underscores the critical need for predictive biomarkers to identify which patients are most likely to benefit from this combination therapy. While our study design did not assess biomarkers, factors such as PD-L1 expression, tumor mutational burden (TMB), and human papillomavirus (HPV) status are known to influence immunotherapy response in HNSCC and should be a focus of future research. Furthermore, our subgroup analysis revealed that advanced age (≥60 years) was associated with a significantly higher risk of death (HR = 4.17). This could be due to reduced tolerance for aggressive combination therapy, age-related decline in immune function (immunosenescence), or a higher burden of comorbidities. This finding suggests that a more cautious, individualized approach is needed for elderly patients.

This study has several limitations. First, its retrospective, single-center design carries an inherent risk of selection bias and limits the generalizability of the findings. Although we used PSM to balance baseline characteristics between the two groups, this method can only account for measured confounders, and unmeasured variables may still influence the results. Second, the sample size is relatively small (89 patients in each matched cohort), which may limit the statistical power to detect a significant difference in 3- and 5-year OS rates. Third, key prognostic data, such as patient HPV status (a major factor in oropharyngeal but less so in oral cancer) and PD-L1 expression levels, were not universally available for analysis. Furthermore, the lack of Disease-Free Survival (DFS) or Progression-Free Survival (PFS) data limits our ability to evaluate the impact of PD-1 inhibitors on early tumor relapse and long-term disease control. The etiology of the oral cancer (e.g., smoking, alcohol use) could also affect outcomes but was not stratified in the analysis. Fourth, there may have been heterogeneity in the specific chemotherapeutic agents and radiotherapy techniques used over the study period that could not be fully controlled for.

## Conclusion

In conclusion, this study provides compelling evidence that the addition of a sequential PD-1 inhibitor to standard chemoradiotherapy offers a significant overall survival benefit for patients with oral cancer, manifested as a significantly reduced risk of death and superior median OS. Despite an increased rate of manageable adverse events, the combination regimen appears to have a favorable risk-benefit profile. The particular benefit observed in patients receiving concurrent chemoradiotherapy highlights potential synergistic effects. However, the lack of statistical significance for 3- and 5-year landmark OS rates, coupled with the study’s retrospective nature and limited sample size, underscores the need for cautious interpretation. These promising findings strongly support the need for further validation in larger, multi-center, prospective, randomized controlled trials to definitively establish the efficacy of this combination, optimize patient selection through biomarker analysis, and solidify its place in the standard of care for oral cancer.

## Data Availability

The raw data supporting the conclusions of this article will be made available by the authors, without undue reservation.
