# Supplementary material for: Sequential PD-1 inhibitor after adjuvant radiotherapy for postoperative oral cancer: a propensity score matching retrospective cohort study
Source: Front Oncol. 2026 May 14;16:1798895. doi: 10.3389/fonc.2026.1798895 (PMC13215798; doi:10.3389/fonc.2026.1798895)
Supplement: Supplementary file 1 [file Table1.doc]

**Supplementary Material**

**Table S1.** Specific PD-1 inhibitors used before and after propensity score matching.

**Table S2** Univariable and multivariable for overall survival adjusted for lifestyle factors (smoking, alcohol, and betel nut use) in the unmatched cohort.

**Table S3.** Univariable and multivariable for overall survival adjusted for lifestyle factors (smoking, alcohol, and betel nut use) in the matched cohort.

**Table S1 Specific PD-1 inhibitors used before and after propensity score matching.**

| PD-1 inhibitor | Pre-PSM, n (%) | Post-PSM, n (%) |
| --- | --- | --- |
| Zimberelimab | 66 (52.8) | 48 (53.9) |
| Camrelizumab | 43 (34.4) | 30 (33.7) |
| Sintilimab | 8 (6.4) | 6 (6.7) |
| Tislelizumab | 3 (2.4) | 2 (2.2) |
| Serplulimab | 3 (2.4) | 2 (2.2) |
| Toripalimab | 1 (0.8) | 1 (1.1) |
| Penpulimab | 1 (0.8) | 0 (0.0) |
| Total | 125 (100) | 89 (100) |

Abbreviations:PSM, propensity score matching

**Table S2 Univariable and multivariable for overall survival adjusted for lifestyle factors (smoking, alcohol, and betel nut use) in the unmatched cohort.**

| Parameter | Univariable HR (95% CI) | Univariable P value | Multivariable HR (95% CI) | Multivariable P value |
| --- | --- | --- | --- | --- |
| Treatment  (PD-1 inhibitor vs. control) | 0.44 (0.27-0.73) | 0.001 | 0.57 (0.33-0.98) | 0.042 |
| Age  (<60 vs. ≥60) | 1.17 (0.78-1.77) | 0.438 | 0.94 (0.58-1.54) | 0.807 |
| Sex  (Female vs. Male) | 1.44 (0.92-2.24) | 0.107 | 1.5 (0.88-2.56) | 0.137 |
| ECOG  (1 vs. 0) | 1.56 (1.04-2.36) | 0.033 | 1.35 (0.81-2.23) | 0.25 |
| T Stage  (1&2 vs. 3&4) | 1.21 (0.80-1.82) | 0.362 | 1.18 (0.78-1.78) | 0.443 |
| N Stage  (0&1 vs. 2&3) | 1.64 (1.09-2.47) | 0.018 | 1.69 (1.10-2.60) | 0.017 |
| Chemo  (Present vs. Absent) | 0.60 (0.39-0.92) | 0.019 | 0.66 (0.42-1.04) | 0.075 |
| Pathological differentiation (G1 vs. G2-G3) | 0.92 (0.61-1.40) | 0.699 | 0.79 (0.50-1.24) | 0.307 |
| Margin  (Negative vs. Positive) | 0.92 (0.57-1.49) | 0.739 | 0.85 (0.51-1.39) | 0.507 |
| PNI  (Present vs. Absent) | 0.81 (0.53-1.22) | 0.315 | 0.66 (0.36-1.21) | 0.18 |
| LVI  (Present vs. Absent) | 0.97 (0.63-1.51) | 0.904 | 1.22 (0.64-2.36) | 0.546 |
| Smoking history  (Ever vs. Never) | 0.8 (0.53-1.21) | 0.286 | 1.14 (0.66-1.99) | 0.636 |
| Alcohol intake  (Ever vs. Never) | 0.82 (0.51-1.31) | 0.409 | 0.94 (0.53-1.67) | 0.827 |
| Betel nut habit  (Ever vs. Never) | 0.25 (0.06-1.02) | 0.053 | 0.37 (0.09-1.58) | 0.179 |

Abbreviations: ECOG, Eastern Cooperative Oncology Group; T stage, tumor stage; N stage, node stage; Chemo, Chemotherapy; G, grade; margin, margin status; PNI, perineural invasion; LVI, lymphovascular invasion; HR, hazard ratio; CI, confidence interval.

**Table S3. Univariable and multivariable for overall survival adjusted for lifestyle factors (smoking, alcohol, and betel nut use) in the matched cohort.**

| Parameter | Univariable HR (95% CI) | Univariable P value | Multivariable HR (95% CI) | Multivariable P value |
| --- | --- | --- | --- | --- |
| Treatment  (PD-1 inhibitor vs. control) | 0.56 (0.29-0.93) | 0.018 | 0.58 (0.31-0.96) | 0.038 |
| Age  (<60 vs. ≥60) | 1.58 (0.95-2.64) | 0.081 | 1.14 (0.58-2.23) | 0.698 |
| Sex  (Female vs. Male) | 0.79 (0.41-1.52) | 0.477 | 1.41 (0.65-3.05) | 0.385 |
| ECOG  (1 vs. 0) | 1.42 (0.85-2.37) | 0.176 | 1.25 (0.64-2.42) | 0.516 |
| T Stage  (1&2 vs. 3&4) | 1.48 (0.89-2.48) | 0.131 | 2.13 (1.16-3.91) | 0.015 |
| N Stage  (0&1 vs. 2&3) | 1.33 (0.79-2.22) | 0.28 | 1.06 (0.58-1.94) | 0.842 |
| Chemo  (Present vs. Absent) | 0.65 (0.39-1.08) | 0.097 | 0.54 (0.30-0.97) | 0.038 |
| Pathological differentiation (G1 vs. G2-G3) | 0.72 (0.43-1.20) | 0.211 | 1.04 (0.56-1.93) | 0.896 |
| Margin  (Negative vs. Positive) | 0.87 (0.47-1.61) | 0.66 | 0.53 (0.25-1.11) | 0.091 |
| PNI  (Present vs. Absent) | 0.83 (0.50-1.40) | 0.49 | 0.94 (0.43-2.07) | 0.884 |
| LVI  (Present vs. Absent) | 0.94 (0.55-1.62) | 0.831 | 1.05 (0.46-2.37) | 0.908 |
| Smoking history  (Ever vs. Never) | 0.99 (0.57-1.70) | 0.961 | 1.18 (0.55-2.52) | 0.67 |
| Alcohol intake  (Ever vs. Never) | 1.19 (0.67-2.12) | 0.557 | 1.19 (0.57-2.44) | 0.646 |
| Betel nut habit  (Ever vs. Never) | 0.4 (0.10-1.64) | 0.202 | 0.44 (0.10-1.98) | 0.285 |

Abbreviations: ECOG, Eastern Cooperative Oncology Group; T stage, tumor stage; N stage, node stage; Chemo, Chemotherapy; G, grade; margin, margin status; PNI, perineural invasion; LVI, lymphovascular invasion; HR, hazard ratio; CI, confidence interval.
